# Towards a model for integrative medicine in Swedish primary care

**DOI:** 10.1186/1472-6963-7-107

**Published:** 2007-07-10

**Authors:** Tobias Sundberg, Jeremy Halpin, Anders Warenmark, Torkel Falkenberg

**Affiliations:** 1 Unit for Studies of Integrative Health Care, Department of Neurobiology, Care Sciences and Society, Division of Nursing, Karolinska Institutet 23300, 141 83 Huddinge, Sweden; 2 Axelsons Gymnastiska Institut, Gästrikegatan 10-12, 113 82 Stockholm, Sweden; 3 Rågsveds Husläkare, Kumlagatan 15, 124 65 Bandhagen, Sweden

## Abstract

**Background:**

Collaboration between providers of conventional care and complementary therapies (CTs) has gained in popularity but there is a lack of conceptualised models for delivering such care, i.e. integrative medicine (IM). The aim of this paper is to describe some key findings relevant to the development and implementation of a proposed model for IM adapted to Swedish primary care.

**Methods:**

Investigative procedures involved research group and key informant meetings with multiple stakeholders including general practitioners, CT providers, medical specialists, primary care administrators and county council representatives. Data collection included meeting notes which were fed back within the research group and used as ongoing working documents. Data analysis was made by immersion/crystallisation and research group consensus. Results were categorised within a public health systems framework of structures, processes and outcomes.

**Results:**

The outcome was an IM model that aimed for a patient-centered, interdisciplinary, non-hierarchical mix of conventional and complementary medical solutions to individual case management of patients with pain in the lower back and/or neck. The IM model case management adhered to standard clinical practice including active partnership between a gate-keeping general practitioner, collaborating with a team of CT providers in a consensus case conference model of care. CTs with an emerging evidence base included Swedish massage therapy, manual therapy/naprapathy, shiatsu, acupuncture and qigong.

**Conclusion:**

Despite identified barriers such as no formal recognition of CT professions in Sweden, it was possible to develop a model for IM adapted to Swedish primary care. The IM model calls for testing and refinement in a pragmatic randomised controlled trial to explore its clinical effectiveness.

## Background

Complementary therapies (CTs) can be defined as various medical and health care practices, products or systems not currently considered as part of conventional medicine in general or of a country's own medical tradition in particular, or not fully integrated into that country's dominant health care system [1,2]. Authors have reported increased public use and recognition of CTs in western societies during the last decades [3,4]. Not only patients but also health care providers and allies have changed perspectives in relation to CTs. This may be illustrated by the increased integration of CTs into conventional medical settings, health care organisations and insurance plans [5-7], the increased number of medical training programs that are including courses on CTs and IM [8,9], as well as academic centres and hospitals integrating selected CTs into their services and research [10,11]. These trends indicate a narrowing of the gap between previously opposing domains, possibly as a combined result of consumer pressure and political will on the one hand, and emerging evidence of effectiveness, safety concerns and normative recommendations, e.g. by the World Health Assembly [12], on the other.

A similar trend has been noted in Scandinavia and Sweden [13,14]. In 2000, the newly founded Stockholm county council CT center [15,16] commissioned a study on the public use and recognition of CTs in Stockholm county [16]. Recognising an increased popularity and utilisation of CTs, these results contributed to strategic financial support for CT education of selected health care professionals employed by Stockholm county. A Swedish national survey of CT provision in county councils reported that some CTs, i.e. massage and acupuncture, were provided in all 16 county councils [17]. About half or more of the county councils offered an additional range of CTs, provided in various ways by diverse conventional and CT practitioners [17]. Parallel to these findings, student demand contributed to the first academic CT courses and the establishment of an academic CT center, i.e. the Unit for Studies of Integrative Health Care (formerly the Center for Studies of Complementary Medicine) at Karolinska Institutet.

Emerging evidence indicating effectiveness has been described for some CTs, e.g. massage, acupuncture, manual therapy and exercise, in the management of common primary care diagnoses such as low back pain, neck pain or headache [18-21]. However, the diversity in terms of CT modalities, modes of delivery, and the degree of legitimacy and acceptance (or lack thereof) that CTs are afforded in various national policies, reveal that commonly accepted working definitions and terms are lacking, as well as agreed policies on how various CTs might be applied or integrated in the management of common medical conditions [12,22-24]. Integrative medicine (IM) can be defined as an attempt to combine conventional medical therapies with more evidence-based CTs [1]. It has also been pointed out that the concept of IM should not simply mean adding CTs to conventional care. IM models should emphasise health and healing rather than disease and symptomatic treatment, and may include biomedical as well as complementary social, psychological and – when relevant – even spiritually oriented interventions [25]. Currently, there is a relative lack of consensus regarding IM as well as a lack of research evidence supporting different IM models delivering CTs in mainstream medical settings.

To contribute towards filling this gap in knowledge, we conducted an IM pilot project in three phases, i.e. the development, implementation and evaluation of an IM model adapted to Swedish primary care. This article describes results from the development and implementation phases.

## Methods

### Study design and setting

In order to develop and implement an IM model adapted to Swedish primary care, we employed a qualitative, inductive study design, utilising explorative, working group methods with a pragmatic problem-based approach. The IM project was conducted in the clinical setting of a primary care unit in Skarpnäck during 2003–2006. Skarpnäck is a suburban area south of Stockholm that to a certain extent can be characterised by having a population with a socio-economic status marked by higher rates of unemployment and sick leave, and of more people with lower incomes and on welfare support, compared to the average levels in Stockholm [26].

### Participants and procedures

The research group that developed and implemented the IM model included a senior researcher, a doctoral student, an IM provider group consisting of a specialist general practitioner and eight senior CT providers (three Swedish massage therapists, a manual therapist/naprapath (a naprapath is a common provider of manual therapy in Sweden working with techniques like spinal mobilisation/manipulation, stretching and massage), two shiatsu therapists, an acupuncturist and a qigong therapist), two research assistants and a CT assistant. Participants were mainly recruited through the Unit for Studies of Integrative Health Care at Karolinska Institutet, e.g. were made up of lecturers and staff affiliated with the academic CT courses. Investigative procedures involved regular research group meetings, developing the IM model on site at the primary care unit in Skarpnäck. Group meetings were initially held once every one to two months, and at later stages of implementation of the IM model, once every two to three weeks. To describe additional perspectives on CTs/IM, the research group meetings were complemented with group and key informant meetings with local CT providers, medical specialists, primary care unit administration/management and county council representatives. These stakeholders were mainly recruited through the county council CT center, local primary care units and research group members' clinical and educational affiliations. During the progression of the IM project a total of about forty meetings and case conferences were held.

### Data collection, analysis and categorisation

The first author (TS) was mainly responsible for collecting data during research group meetings in the form of meeting notes, which were then submitted for feedback, discussed within the research group, and used as ongoing working documents. Additional data collection included individual field notes from seminars and meetings, and written material such as handouts and research articles, as well as the practical experience gained from implementing the IM model. The pooled data were analysed by means of immersion/crystallisation [27] and research group consensus. Immersion/crystallisation is a methodological strategy suitable for clinical primary care research, entailing repetitive cycles of data collection, data analysis, reflection and refinement of strategies, followed by further data collection. A consensus approach, arrived at by participatory input from the research group, was utilised to develop pilot project strategies and finalise the proposed case management model for IM adapted to Swedish primary care presented in this paper.

Clarifying relationships between different components of a public health system, has been suggested as an important step towards providing a scientific basis for the study of public health system performance [28]. Hence, key project findings from the IM project have been categorised in terms of a public health systems framework approach, comprised of processes (i.e. research group activities), structures (i.e. organisational elements created by the research group), and outcome (i.e. the clinical IM model adapted to Swedish primary care) [28,29].

The research project was approved by the regional ethics committee at Karolinska Institutet (Dnr: 668-03, 650-04, 121-32).

## Results

### Processes

**Figure 1:**
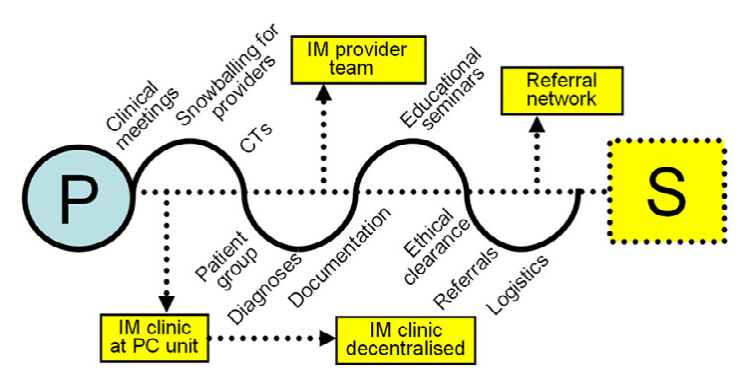
**Processes and structures**. Key processes (P), i.e. research group activities, and structures (S), i.e. organisational elements, created by the research group in the development and implementation of the integrative medicine model adapted to Swedish primary care.

The idea to develop an IM model adapted to Swedish primary care originated in an extended informal dialogue among course leaders, students and lecturers affiliated with the academic CT courses at the Unit for Studies of Integrative Health Care. The dialogue was fuelled by some of the participants' international clinical experience of providing conventional care and CTs, as well as evidence of increased utilisation of CTs, and the documented desire for increased collaboration and research on the part of the citizens of Stockholm county [16]. During 2003–2004, sufficient number of interested parties emerged to be able to initiate an IM research group, with a network of potential providers willing to consider participating in a clinical trial. A fully financed doctoral student (TS) became project coordinator and the head of the Unit for Studies of Integrative Health Care (TF) became principal investigator of the IM research project. The key processes of the project have been outlined in Figure 1.

A snowballing process to identify further team members started focusing on recruiting CT providers rooted within their respective medical models and with experience of sharing cases with conventional providers in order for them to be able to actively take part in the mutual exchange and learning process engaged in by the group. As a result, senior practitioners were included in the IM provider team. In this process, we identified the most suitable CTs to be included as part of the IM model. Globally, we found that the combinations of therapies and providers involved in different IM approaches to health care delivery could be highly diverse. In addition to an emerging evidence base of safety and effectiveness, it was reasoned that context-dependent elements such as available resources, funding, reimbursement and legal issues as well as popularity, utilisation patterns and potential referral pathways might be among the important determinants in the selection process.

Subsequent clinical group meetings aimed at training the selected IM provider team to work collaboratively, utilising a consensus case conference model within the primary care setting. During this period, meetings would typically feature professional presentations and educational items on issues relating to the philosophy and theory of different medical models, and the practice of conventional primary care and selected CTs. The main discussion themes covered conventional and CT perspectives on practice patterns and case management strategies, including approaches to diagnosis, treatment, prevention and documentation. Stakeholder perspectives on facilitators, barriers and strategies for developing and implementing an IM model in Swedish primary care were also explored and a summary of findings have been provided in Table 1. As the IM model started to crystallise, the research group approached primary care providers in the area to inform about the IM project and to investigate possibilities for a referral network for the upcoming IM trial. Briefly, this was achieved by the general practitioner of the research group heading informal, in-house presentations at the primary care units of Skarpnäck and Bagarmossen supplemented by seminars, together with the group coordinator, at three neigbouring primary care units. A total of about 20–30 primary care staff members, primarily representing general practice, but also orthopaedics, neurology, surgery, physical therapy and management/administration, were introduced to the IM project in this way and different views of the IM project were discussed. Additionally, the group participated in two information meetings with representatives from Stockholm county council targeting conventional care and CTs. After the meetings, dialogue continued with interested parties. It is beyond the scope of this article to give a full presentation of findings related to these meetings. However, Table 1 provides a summary of conventional care stakeholder perspectives on IM that were largely derived from these meetings and dialogues.

**Table 1:** Stakeholder perspectives. Summary of conventional care (CC), complementary therapies (CT) and research (RES) stakeholder perspectives on facilitators, barriers and strategies for developing and implementing a model of integrative medicine (IM) in Swedish primary care.

**Stake- holder**	**Facilitators**	**Barriers**	**Project strategies**
**CC**	Documented public desire for increased collaboration	Lack of knowledge and know-how	General practitioner gatekeeper with CT interest, knowledge and experience leading the clinical part
	Limitations of conventional care in certain areas/cases	Primary care unit resources	General practitioner meetings with management/administration about resource allocation and logistics
	Personal interest to provide more holistic primary care	No formal IM recognition in Sweden	Priority of reimbursing CT providers
	Improve knowledge and evidence base of IM	Scientific evidence base	Part-time provider commitment
	Improve recognition of IM	Large variation of CT terminologies and documentation routines	Ethical clearance
		The Swedish Health Services Act	

**CT**	Increase respect for patients' treatment choices	Value added tax (25%) on CTs and no public insurance policy for CTs	CT providers with experience sharing cases with conventional providers
	CT access to interdisciplinary cooperation	No official recognition of CT professions	An IM model broad enough to encompass all selected CTs/medical models
	Represent different medical models within Swedish primary care	Interdisciplinary dialogue rare	Consensus case conferences to facilitate and document interdisciplinary dialogue
	Extend the evidence-based medicine concept	Unfamiliarity with primary care documentation routines	Part time CT provider commitment
	Improve national awareness and recognition of existing international IM practices	The Swedish Health Services Act	Include quality of life, stress and wellbeing outcomes
	Improve focus on care, health promotion and Prevention		

**RES**	Explore stakeholder perspectives on IM in Swedish primary care	Limited evidence base for IM	Initial core group development meetings to facilitate research project
	Explore patient experiences of integration of complementary therapies in primary care	Lack of published randomised clinical trials of IM in primary care	Include both qualitative and quantitative methods of evaluation
	Explore general clinical effectiveness of the IM model vs. treatment as usual	Difficulties to obtain research funding	Information and educational seminars to improve understanding between stakeholders and facilitate shared documentation routines
	Improve the evidence base for integration of CTs into primary care	Unknown recruitment speed and recruitment pattern of patients	Continuous grant writing to secure funding
		No pre-defined or given set of outcomes	Referral network of primary care units
		No established referral network	

Another team process consisted of identifying which patients and diagnoses to focus on for the pilot trial. The research group reasoned that most CTs provided by the IM team commonly dealt with various types of sub-acute to chronic pain syndromes, mainly in the musculoskeletal field. Considering CTs commonly used in Stockholm county [16], and the feasibility for inclusion in a clinical pilot trial in the primary care setting, the IM model was finally set to include patients consulting their general practitioner with low back pain or neck pain, with or without headache, for a duration of at least two weeks. Patients with diagnosed pathological changes such as fractures or ruptured discs, malignant disease, psychiatric diesease, or those not proficient in spoken and written Swedish were excluded.

As ethical approval had been granted and the IM trial came closer to implementation, research group meetings would to a greater extent deal with logistical aspects, including the practical set-up of the IM clinic at the Skarpnäck primary care unit. Localities and room allocations were determined, access hours specified, patient documentation, booking and referral procedures defined. Here the project coordinator and the general practitioner of the research group worked together with the primary care administration/management. When the IM trial had started, the research group meetings continued, combined with consensus case conferences conducted in order to provide patient care in the IM model.

### Structures

The main structure created was an IM clinic set up as an operative centre five days per week, on site at the primary care unit in Skarpnäck. The IM clinic housed all major group activities as well as the provision of conventional and CT consultations and treatments. Unforeseen contract changes while renegotiating the rental lease with the property owner forced the IM clinic to move into a new building nearby. At the new site, the IM clinic had to share offices with other concurrent conventional primary care activities, leaving only certain days per week available for IM activities. This resulted in a new mode of operation that lasted thoughout the IM project, where CT consultations and treatments where additionally decentralised to the clinics of the participating CT providers for feasibility reasons.

A second important structure was setting up the team providing treatments for patients in the IM model. It was considered important for both individual and group-based self-help activites to be part of the CTs provided by the IM team, as these represented complementing aspects of care. Initially, both yoga and qigong were considered as group-based therapies. However, for practical, logistical and funding reasons, finally, only qigong was included. The IM provider team finalised crystallised as consisting of one general practioner and eight CT providers, representing Swedish massage therapy, manual therapy/naprapathy, shiatsu, acupunture and qigong. Hence, both individual and group-based CT approaches were included. All the providers commited to the IM project on a part-time and partial reimbursement basis.

The final element was the primary care referral network for recruiting patients. Here general practitioners from four primary care units in the same geographical area of south-suburban Stockholm were involved. The key structures have been outlined in Figure 1.

### Outcomes

**Figure 2:**
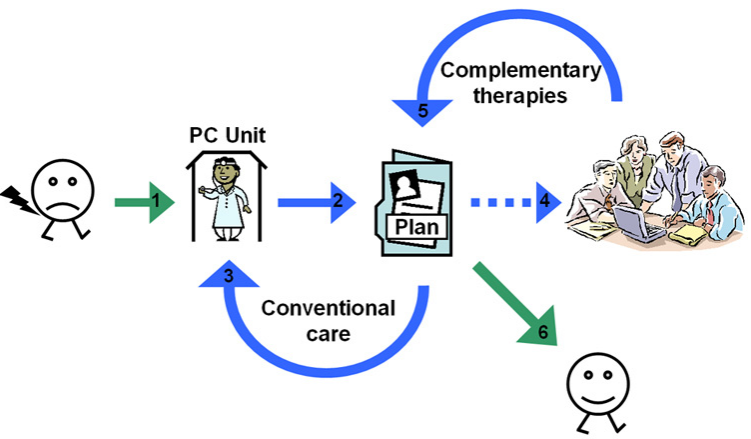
**Outcome, the integrative medicine model**. The integrative medicine model adapted to Swedish primary care illustrated as a clinical case management flowchart: 1) The patient with sub-acute to chronic low back pain or neck pain consults the general practitioner gatekeeper at the primary care unit.; 2) The patient and the general practitioner develop a treatment plan.; 3) The patient is offered conventional care, i.e. treatment as usual.; 4) Should complementary therapies be considered appropriate, these are integrated into the treatment plan by way of a consensus case conference with the integrative medicine provider team.; 5) The patient is offered complementary therapies as part of the treatment plan, i.e. integrative care.; 6) When the treatment plan is completed the case management is finished. Please note that integrative care was only delivered for up to 12 weeks.

The IM model (Figure 2) was aimed towards delivering a patient-centred mix of conventional and complementary medical solutions in the individual case management of patients with subacute to chronic, low back pain or neck pain. It was characterised by the active partnership of a general practitioner with knowledge of CTs, and a team of selected CT providers with knowledge of biomedicine. The general practitioner served as the gatekeeper with the overall responsibility for the medical management of the patient in the IM model, i.e. in accordance with the Swedish regulatory framework for providing conventional primary care. This included developing a conventional treatment plan, e.g. the ordination of sick-leave and prescription drugs, in dialogue with the patient as well as discussing the possible integration and appropriateness of selected CTs in the management of individual cases. Should an integration of CTs be considered appropriate, these were integrated into the treatment plan by way of a consensus case conference with the IM provider team. During the consensus case conference, the general practitioner and the CT providers engaged in a roundtable discussion, identifying appropriate treatment strategies integrating selected CTs, tailored to the patient's needs and concerns. As active CT treatments commenced, regular consensus case conferences followed, combining conventional and complementary clinical reasoning with a non-hierarchical, open, continuous and parallel interchange of ideas, in order to verify and improve the ongoing clinical management of the patient. For the IM trial, this involved up to 10 treatments over a period of no more than 12 weeks. Patients did not participate in the consensus case conferences, as it was considered more efficient for them to take part in the health care process by way of intermittent personal interaction with the IM provider team. In the IM model, treatments were documented in the providers' case records as well as in a mutual IM record for each patient. Fees for CT consultations and treatments in the IM model were set as non-reimbursed patient fees comfortably adapted to conventional primary care fee levels, i.e. on average 5 EUR per treatment. In order to resemble an insurance policy, no additional payment was asked of patients after six CT consultations or treatments.

## Discussion

The methodological approaches of immersion/crystallisation and research group consensus were selected, based on the aim of multiprofessional interaction and mutual decision-making in developing and implementing a comprehensive model for IM in Swedish primary care. Immersion/crystallisation has been used in previous research on the integration of CTs in primary care [30]. Alternative methods for developing an IM model could have been based on e.g. the Delphi technique, interviews with expert providers or simply replicating other IM models described internationally. However, considering that IM was a new and unexplored field in Sweden, we were not able to draw upon previous results or clinical experiences, hence the interactive group process involving team meetings and achieving research group consensus was regarded as being more relevant. Joint group activities were carried out with the aim to facilitate a spirit of mutual understanding and cooperation towards developing and "owning" the proposed IM model on as equal terms as possible, considering Swedish rules and regulations.

The regular team meetings evolved as a key factor bridging academic, clinical and CT provider perspectives on IM. The cross-fertilisation of ideas and perspectives within the research group during these meetings were important for developing the IM model. The participants viewed these processes as highly educational and relevant in terms of facilitating an increased mutual understanding and exploring potential areas for the development of the IM model. It was uncommon for participants to have experienced such interactive dialogues elsewhere. Notably, the lack of dialogue and communication has been described as barriers towards implementing IM [31,32]. Arguably, the fact that all our providers, despite their different backgrounds, shared a basic knowledge of biomedicine, may have contributed to the atmosphere of respectful sharing of various views on the patient cases that emerged. However, the large variation in terminologies and documenting routines between conventional and CT providers was perceived as a barrier in the development of common procedures for the clinical documentation of IM care. An important factor facilitating this process was certainly their improved understanding of each other's medical models, gained through the group seminars and lectures. A published case study of communication has shown that IM panel members representing a wide range of theories of health and healing were able to communicate easily with one another, when they limited themselves to the scientific language of biomedicine [33].

The partial decentralisation of the IM clinic, although not initially planned for, was a change that turned out to facilitate the participation of CT providers in the IM project, chiefly by not having to split their days between different locations. This change also demonstrated the potential flexibility of the IM structure, whether as part of a primary care unit or as a cohesive network of conventional and CT practices. It has been reported that few IM models have proven to be sustainable [34]. This type of decentralised setup however required a minimum of resources to be sustained. Another aspect of decentralisation might be less informal interaction among providers, e.g. in between seeing patients.

Current Swedish law, ie The Professional Activity in the Health Services Act (1998:531) (Lagen om yrkesverksamhet på hälso- och sjukvårdens område) [35] holds that medical and therapeutic approaches provided within the conventional health care sector should be based on, and adhere to, evidence from both tested experiential knowledge from clinical practice, and scientific research. Accordingly, for any therapy, complementary or conventional, to be considered for provision within the Swedish health care system, at least theoretically, it must have the support of clinical practice in managing a given condition, produce effects that can be explained in biomedical terms, and be supported by research evidence in favour of the application. In terms of generalisability of the IM model, other countries may have different approaches with regard to legal frameworks which prohibit, tolerate or even promote integration of CTs into conventional health care settings, where the latter condition might be exemplified by recent Norwegian health law reform. Norway's new legislation promotes enhancing collaboration between conventional medicine and CTs [36,37]. The CTs of the IM model at least had support from clinical practice and an emerging evidence base in general [18-21,38,39]. Clinical biomedical reasoning around how the selected therapies in the IM model could complement each other was regarded as valuable. For example, massage (Swedish and shiatsu) and stretching target muscle function; manual therapy/naprapathy target joint function; acupuncture/acupressure target pain modulation and qigong targets physical exercise and home treatment. Arguably, the selected therapies could also have parallel explanations of interaction, originating in their original philosophical/cultural/medical domains, e.g. traditional Chinese medicine and the five element theory. The latter perspectives may be facilitating factors for IM by certain providers and patients alike, seeking a broader view of the philosophy, art and science of the selected therapeutic approaches. It has been reported that patients use CTs because they find such health care alternatives to be more congruent with their own values, beliefs and philosophical orientations [40]. The selection of CTs for the IM model might be argued to lack some perspectives, e.g. nutritional and mental health aspects. However, besides over-the-counter food supplements and natural remedies, such CTs have not been represented among the most prevalent CTs in recent Swedish population-based survey findings [13,16].

Previously, Swedish users of CTs have been shown to be predominantly well-educated, middle/high income groups [13,16]. Considering the socio-economic situation at the location for implementing the IM model, i.e. the Skarpnäck area in south suburban Stockholm, the IM project aimed to provide a population with IM living in an area with limited access to CTs, due to e.g. financial reasons [26]. Potentially a barrier for implementing the IM model, this meant testing the IM model in an area of lower socioeconomic status. Related to this, it was considered facilitating to have a low fee-per-CT treatment and a low maximum treatment cost to obtain all CT treatments in the pilot randomised controlled trial. This was preferred over having no fees at all, as a small investment was reasoned to commit the patients to follow the recommended treatment programme. However, we do not know how this financial setup might be transferable to the clinical situation in Sweden today, since patients currently pay for CTs out-of-pocket. Few private insurance companies in Sweden provide compensation for CT services compared to the situation in other high income countries, e.g. the US [5,6]. Another barrier related to implementing our IM model was that the participants of the IM provider group were initially not reimbursed for their services, and even later, after external funding was awarded, at significantly lower levels than their ordinary fees.

General practitioners have previously been proposed as gatekeepers in research on IM [30]. However, in contrast to an IM model that may rely on referrals by the general practitioner in order for the patient to access specific integrated CTs [30,41], the proposed IM model can be characterised as aiming for interdisciplinary, non-hierarchical decision making involving a mix of conventional and complementary medical solutions to individual case management. Although aiming for all providers in the proposed IM model to be hierarchically on the same level, practically and clinically, the general practitioner had to be the overall gatekeeper in charge. The IM provider team did not express any negative sentiments as to how this influenced the team setup and functioning. In Sweden, only licensed medical doctors are permitted to fully utilise the complete range of medical services, referrals and ordinations of sick leave for their patients. Nevertheless, the question of who should be the gatekeeper might justifiably be regarded as a controversial issue, as the possibility for other professions to be gatekeepers might be considered in other countries, provided an appropriate legal framework allows for this.

The importance of consensus has been suggested as being more significant the further a health care practice model moves towards integrative care [22]. We see the group-based consensus approach of the proposed IM model as having several potential advantages over parallel or referral models of IM [22,30,41]. Hypothetically, such advantages might include providing an IM model with increased team-building and cross-fertilisation of ideas, which could lead to increased diagnostic and therapeutic capacities, increased safety cross checking, decreased risk for negative treatment interactions, increased sharing of resources, reduced length of treatment cycles, reduced number of revisits in primary care, and improvement of health care cost-effectiveness by reducing health care costs, including cost of drugs. However, such bold comparative advantages and dramatic benefits remain to be explored further.

Lastly, the development and implementation of a participatory consensus IM model suitable on the one hand to testing in a randomised control trial, and on the other hand applicable to the routines of regular Swedish primary care, was both time-consuming and challenging. This is in line with what has been described by Norwegian and Danish researchers [36]. A summary of general lessons learned from our IM project and some future recommendations for IM in Swedish primary care are described in Table 2.

**Table 2:** Lessons learned and future recommendations. Summary of general lessons learned and future recommendations from developing and implementing a model of integrative medicine (IM) in Swedish primary care.

**General lessons learned**	**Future recommendations**
It was possible to develop a model for IM adapted to Swedish primary care despite various identified barriers.	Funding and resource allocation beforehand important to improve provider participation and planning.
Both a centralised and a decentralised clinic possible for delivering IM in primary care, the latter requiring less primary care unit resources.	Health economic evaluation of IM management vs. treatment as usual needed to motivate management decision.
Time and funding are essential to enable staff commitment, routines and resources as within normal primary care practice.	Availability of general practitioners' specialist training in IM important.
Need for a general practitioner with complementary therapy interest, knowledge and/or experience to coordinate the IM provider group.	Common IM documentation should reflect multi-modular management, and preferably be computer-based.
IM case management slightly more time consuming, but improved case conference experience contributed to more efficient case management.	Combination of qualitative and quantitative research methods useful.
Continuing seminars and discussions can improve understanding, knowledge, motivation and recognition between stakeholders and different medical models. Together with a shared knowledge of basic biomedicine this facilitate interdisciplinary dialogue and collaboration.	
Clinical practice and communication were smooth within the IM group but written documentation procedures were more difficult to standardise.	

## Conclusion

IM is an emerging area of relevance for providers of conventional and complementary care in Sweden. We have described some key findings from the development and implementation of a proposed IM model adapted to the Swedish primary care setting. The IM model builds on active partnership between a gate-keeping general practitioner with an informed knowledge of CT models and practice, having overall medical management responsibilities, collaborating with and coordinating a team of selected CT providers by means of consensus case conferences. The proposed IM model needs testing and refinement in pragmatic, randomised controlled trials before integration into the Swedish primary care system can be recommended.

## Competing interests

Tobias Sundberg and Torkel Falkenberg are affiliated with the Unit for Studies of Integrative Health Care (formerly the Center for Studies of Complementary Medicine) at Karolinska Institutet. Tobias Sundberg was financed by a grant from the Foundation for Manual Therapy Research (Insamlinsstiftelsen för forskning om manuella terapier) during the IM project. Anders Warenmark is a general practitioner with a background in Swedish massage, acupuncture and kinesiology. Jeremy Halpin is an acupuncture and shiatsu therapist in private practice.

## Authors' contributions

All authors contributed to and refined the general research idea. TS drafted the original manuscript and participated in redrafting and rewriting. TF conceived the general research design, critically read the manuscript and participated in redrafting and rewriting. JH and AW critically read the manuscript and added information about the IM project and CT aspects. Draft versions of the article have been circulated for comments, and the participating providers in the IM research group approved the final article.

## Pre-publication history

The pre-publication history for this paper can be accessed here:


